# Cholera

**Published:** 1854-02

**Authors:** 


### CHOLERA.

This plague appears to be again impending over us. Cases of it have occurred on nearly all the vessels that have arrived for some months past, at New Orleans and New York, from the ports of Europe. The disease has never been epidemic in our country during the winter season, but there is reason to apprehend that on the return of warm weather it will again spread from those points.

It is a melancholy truth that the profession is nearly as much at a loss with regard to the treatment of Cholera as it was at the end of the first epidemic. All are agreed that the complaint is manageable in its first stage, but when it has become confirmed Cholera, we have to confess the inefficacy of all the modes of treatment hitherto adopted in the disease. But certain facts of great hygienic value have been established by the successive returns of the epidemic. We think one of these is, that the poison which generates it is portable. We do not think this admits of a question. Another is, that the places visited by it once are those which are most liable to suffer on its return. Not only the towns and villages which have experienced it, but the particular localities in such places, and even the very houses, it is said, are those in which it is now reappearing. A third is, that evacuating such houses and neighborhoods often puts a stop to the disease. Crowding seems to be one of the most efficient of its exciting causes. The obvious policy therefore is, if the plague should appear, to remove from the infected house or district. In all places where the epidemic has prevailed, there have been *foci* where it has manifested peculiar intensity. The inhabitants of such neighborhoods ought to fly at the approach of the pestilence. We have seen the happiest effects, in the country, from dispersing the negroes on a plantation when the disease broke out among them. On sending them into the woods, into temporary sheds, we have repeatedly witnessed the subsidence of the complaint.

It is well ascertained that the night air of infected districts is especially pestilent. Physicians attend the sick during the day with impunity, but nurses and those who sleep in them are exceedingly apt to be attacked. We have had cholera in Louisville repeatedly, but it has always exhibited its chief severity in a few limited districts. It has never been general in the city. Now, it appears to us, nothing can be more rational than to quit those districts, if the plague should again visit them. Filth, undoubtedly, is favorable to the spread of cholera, and we cannot be too much on the alert to remove all such sources of the poison. But we should rely much more upon flight. Removal to a very short distance is often sufficient. When the pestilence visited us last, in the summer of 1851, its ravages were almost wholly confined to the corners of four squares at the end of one of our market houses. All other parts of the city were almost entirely exempt.

